# Pretreatment neutrophil-to-lymphocyte ratio is associated with clinical phenotypes and infection-related mortality in microscopic polyangiitis: the multicentre REVEAL cohort study

**DOI:** 10.3389/fimmu.2026.1948318

**Published:** 2026-10-19

**Authors:** Muneyuki Hatta, Ryu Watanabe, Mayu Shiomi, Yuichi Masuda, Ayana Okazaki, Shogo Matsuda, Takuya Kotani, Tomoki Taniguchi, Shingo Iwasaka, Keiichiro Kadoba, Atsushi Manabe, Kaho Jo, Youhei Fujiki, Hirofumi Miyake, Hideki Oka, Shuji Sumitomo, Wataru Yamamoto, Shinya Nakatani, Akihiro Tsuda, Tetsuya Watanabe, Kazuhisa Asai, Motomu Hashimoto

**Affiliations:** 1 Department of Clinical Immunology, Osaka Metropolitan University Graduate School of Medicine, Osaka, Japan; 2 Department of Internal Medicine (IV), Osaka Medical and Pharmaceutical University, Takatsuki, Japan; 3 Department of Rheumatology and Clinical Immunology, Graduate School of Medicine, Kyoto University, Kyoto, Japan; 4 Division of Clinical Immunology and Rheumatology, Yodogawa Christian Hospital, Osaka, Japan; 5 Department of General Internal Medicine, Tenri Hospital, Tenri, Japan; 6 Department of Rheumatology, Kobe City Medical Center General Hospital, Kobe, Japan; 7 Department of Health Information Management, Kurashiki Sweet Hospital, Kurashiki, Japan; 8 Department of Nephrology, Osaka Metropolitan University Graduate School of Medicine, Osaka, Japan; 9 Department of Metabolism, Endocrinology and Molecular Medicine, Osaka Metropolitan University Graduate School of Medicine, Osaka, Japan; 10 Department of Respiratory Medicine, Osaka Metropolitan University Graduate School of Medicine, Osaka, Japan

**Keywords:** ANCA-associated vasculitis, interstitial lung disease, microscopic polyangiitis, neutrophil-to-lymphocyte ratio, renal failure, infection-related mortality

## Abstract

**Background:**

Microscopic polyangiitis (MPA) remains associated with substantial early mortality despite advances in immunosuppressive therapy, highlighting the need for simple biomarkers that facilitate LR) has been associated with disease activity and prognosis in antineutrophil cytoplasmic antibody-associated vasculitis (AAV), its phenotype-specific significance in newly diagnosed MPA remains unclear. We investigated whether pretreatment NLR is associated with clinical phenotype, disease course, and long-term outcomes in MPA.

**Methods:**

Among 418 patients with MPA enrolled in the multicentre REVEAL cohort between 2005 and 2025, 340 newly diagnosed treatment-naïve patients fulfilling the 2022 ACR/EULAR classification criteria were included. Patients were stratified into tertiles according to pretreatment NLR. Baseline characteristics, temporal outcomes, and survival were compared. Infection-related mortality was further evaluated after stabilised inverse probability weighting (IPW).

**Results:**

Higher pretreatment NLR was associated with greater disease severity, including higher Birmingham Vasculitis Activity Score (*P* < 0.001) and Five-Factor Score (*P* = 0.03), more frequent diffuse alveolar haemorrhage (*P* = 0.015), poorer renal function (*P* = 0.003), and more intensive remission-induction therapy, including methylprednisolone pulse therapy and rituximab. In contrast, interstitial lung disease was more common in patients with lower NLR (*P* = 0.001). Despite these baseline differences, disease activity, glucocorticoid tapering, and cumulative organ damage became comparable after treatment initiation. Pretreatment NLR was not associated with relapse-free survival (*P* = 0.44) but was associated with poorer overall survival (*P* = 0.012) and higher infection-related mortality (*P* = 0.002). After adjustment using IPW, the association with overall survival was no longer statistically significant, whereas the increased risk of infection-related mortality remained significant (HR 3.47, 95% CI 1.32–9.09, *P* = 0.034).

**Conclusion:**

Pretreatment NLR reflected distinct inflammatory phenotypes and was independently associated with infection-related mortality in patients with newly diagnosed MPA. As an inexpensive and widely available marker, pretreatment NLR may have potential as a prognostic biomarker, although further validation is required to establish its clinical utility.

## 1 Introduction

Anti-neutrophil cytoplasmic antibody (ANCA)-associated vasculitis (AAV) is a group of life-threatening inflammatory diseases characterised by pauci-immune necrotising vasculitis affecting predominantly small vessels (1, 2). Among the AAV subtypes, microscopic polyangiitis (MPA) is the most common phenotype in East Asian populations and is characterised by renal involvement, diffuse alveolar haemorrhage (DAH), and interstitial lung disease (ILD) (3). Despite advances in immunosuppressive therapy, MPA remains associated with substantial mortality and irreversible organ damage.

Although disease outcomes have improved with recent therapeutic advances, early mortality remains considerable, largely because of severe infections and organ-threatening disease (4, 5). Early identification of patients at high risk is therefore essential for optimising treatment intensity while minimising treatment-related toxicity. Although several clinical factors have been associated with prognosis, they do not fully capture the marked heterogeneity in disease presentation or reliably identify patients at increased risk of infection-related mortality at disease onset. Therefore, a simple biomarker that can be readily applied in routine clinical practice to facilitate early risk stratification remains an important unmet clinical need.

The neutrophil-to-lymphocyte ratio (NLR) is a simple, inexpensive, and readily available biomarker calculated from routine blood counts (6). It has been associated with disease severity and prognosis in various inflammatory (7–9), cardiovascular (10, 11), and malignant diseases (12, 13). In AAV, elevated NLR has been associated with higher disease activity, renal involvement (5, 14), and increased mortality (5, 15, 16). However, previous studies have mainly included heterogeneous populations comprising multiple AAV phenotypes. Consequently, whether pretreatment NLR reflects distinct MPA phenotypes remains unclear. Its association with infection-related mortality, independent of disease severity and treatment intensity, is also uncertain.

Given the distinct clinical phenotype of MPA, we hypothesised that the clinical significance of pretreatment NLR may differ from that observed in mixed AAV cohorts. Therefore, we investigated whether pretreatment NLR is associated with clinical phenotype, disease severity, disease course, and long-term outcomes, with particular emphasis on infection-related mortality, in patients with newly diagnosed MPA.

## 2 Materials and methods

### 2.1 Study design

This multicentre, retrospective, observational study was conducted using data from the REgistry of Vasculitis patients to establish rEAL-world evidence (REVEAL) cohort, an observational registry of patients with AAV in the Kansai region of Japan (17, 18). The REVEAL cohort includes patients treated at six participating institutions: Osaka Metropolitan University, Osaka Medical and Pharmaceutical University, Kyoto University, Yodogawa Christian Hospital, Tenri Hospital, and Kobe City Medical Center General Hospital. For the present study, we analysed patients with MPA who were registered between May 2005 and May 2025.

Patients were stratified into tertiles according to pretreatment NLR: NLR1 (0.852 ≤ NLR < 4.78), NLR2 (4.78 ≤ NLR < 10.3), and NLR3 (10.3 ≤ NLR ≤ 105). Baseline characteristics, temporal outcomes, treatment patterns, and survival outcomes were compared among the three groups.

### 2.2 Patients

Patients with newly diagnosed, treatment-naïve MPA were eligible for inclusion. MPA was diagnosed according to the Chapel Hill Consensus Conference definitions (19). Patients were excluded if they presented with relapsed disease at study entry, had received glucocorticoids or immunosuppressive therapy before referral, had concomitant autoimmune diseases, lacked pretreatment NLR data, or did not fulfil the 2022 American College of Rheumatology/European Alliance of Associations for Rheumatology (ACR/EULAR) classification criteria (20). Patients who were positive for both MPO-ANCA and PR3-ANCA were included as MPA if they did not fulfil the 2022 ACR/EULAR classification criteria for GPA and fulfilled those for MPA.

This study was conducted in accordance with the Declaration of Helsinki and its subsequent amendments. Informed consent was obtained from all patients at the participating institutions. However, for those who visited Kyoto University or Kobe City Medical Center General Hospital or Osaka Metropolitan University before 2021, the requirement for written informed consent was waived, because the data were anonymised. Instead, consent was obtained through an opt-out process. The study protocol was approved by the ethics committees of all participating institutions, including Osaka Metropolitan University (approval no. 2023-027), Kyoto University (approval no. R1540), Osaka Medical and Pharmaceutical University (approval no. 1529), Tenri Hospital (approval no. C23-11), Yodogawa Christian Hospital (approval no. 2023-070), and Kobe City Medical Center General Hospital (approval no. zh250809).

### 2.3 Data collection

Clinical information was retrospectively extracted from electronic medical records at each participating institution. Baseline demographic and clinical variables included age at treatment initiation, sex, smoking history, the Brinkman Index and comorbidities. Laboratory variables included complete blood counts, serum albumin, lactate dehydrogenase, KL-6, estimated glomerular filtration rate (eGFR), C-reactive protein (CRP), serum IgG, myeloperoxidase (MPO)-ANCA, and proteinase 3 (PR3)-ANCA. Pretreatment NLR was defined as the absolute neutrophil count divided by the absolute lymphocyte count, using values measured before the initiation of any immunosuppressive treatment, including glucocorticoids.

Disease severity was assessed using the 2009 Five-Factor Score (FFS) (21), disease activity using the Birmingham Vasculitis Activity Score (BVAS version 3) (22), and cumulative organ damage using the Vasculitis Damage Index (VDI) (23).

Treatment variables included initial glucocorticoid (GC) dose, methylprednisolone pulse therapy, rituximab, intravenous cyclophosphamide, plasma exchange, avacopan and GC exposure during the first 24 months after treatment initiation.

Clinical outcomes included relapse, all-cause mortality, and infection-related mortality. Relapse was defined as recurrent, worsening, or newly developed disease activity requiring treatment escalation at the discretion of the treating physician. The cause of death was independently assessed by at least three physicians and reported according to the classification reached by consensus. Patients lost to follow-up were censored at the date of their last clinical assessment.

### 2.4 Outcomes

The primary objective of this study was to evaluate the associations between pretreatment NLR and baseline clinical characteristics in patients with newly diagnosed MPA. Secondary outcomes included intergroup differences in GC dose, BVAS, and VDI during the treatment course, as well as relapse-free survival, overall survival, cause-specific mortality, and infection-related mortality.

### 2.5 Statistical analysis

Continuous variables are presented as medians with interquartile ranges (IQRs), whereas categorical variables are presented as frequencies and percentages. Baseline clinical characteristics were compared using the Kruskal–Wallis test or Mann–Whitney U test for continuous variables and Fisher’s exact test for categorical variables. Correlations between continuous variables were assessed using Spearman’s rank correlation coefficient.

Intergroup differences in GC dose, BVAS, and VDI during the treatment course were evaluated using the Kruskal–Wallis test. GC dose was compared at baseline, 3, 6, 12, and 24 months, BVAS at baseline, 6, 12, and 24 months, and VDI at 6, 12, and 24 months. For GC dose and BVAS, a complete-case analysis restricted to patients with data available through 12 months was performed as a sensitivity analysis.

Relapse-free survival and overall survival were estimated using the Kaplan–Meier method and compared using the log-rank test. Because the overall survival curves crossed during follow-up, suggesting potential non-proportional hazards, landmark analyses were additionally performed at 2.5 years, the midpoint of the 5-year follow-up period, to evaluate early and late survival separately. Patients who did not survive to the landmark time were excluded from the post-landmark survival analysis. To assess the robustness of the findings, sensitivity analyses were additionally performed at 2 and 3 years. Crude cause-specific death rates were calculated as deaths per person-year and compared using exact Poisson tests. For infection-related mortality, differences among the NLR groups in the cause-specific hazard were assessed using the log-rank test, with non-infection-related deaths treated as censoring events. In addition, the cumulative incidence function (CIF) for infection-related death was estimated with non-infection-related deaths treated as competing events, and differences among the NLR groups were assessed using Gray’s test (24). To evaluate the robustness of the tertile-based findings, we performed a sensitivity analysis using a univariable cause-specific Cox proportional hazards model in which pretreatment NLR was treated as a continuous variable. Given the wide range of NLR values and the presence of extreme values in the upper tail, NLR was log_10_-transformed before modelling. Based on previous reports of a non-linear relationship between NLR and mortality, log_10_-transformed NLR was modelled using a restricted cubic spline with three knots to allow for potential non-linearity on the transformed scale. The overall association and non-linearity were evaluated using Wald tests. The adequacy of the functional form for NLR was assessed using martingale residuals, and the proportional hazards assumption was assessed using scaled Schoenfeld residuals.

Patients with higher pretreatment NLR were older, had greater disease activity, and received more intensive remission-induction therapy. Therefore, inverse probability weighting (IPW) was performed to address measured differences among the NLR groups. The target estimand was the association between pretreatment NLR and the cause-specific hazard of infection-related death after accounting for differences in treatment intensity (25). Propensity scores were estimated using multinomial logistic regression. Guided by the hypothesised causal structure represented in the DAG (**Supplementary Figure 1**), the propensity-score model included age, baseline BVAS, initial GC dose, methylprednisolone pulse therapy, rituximab use, and intravenous cyclophosphamide use. To assess the robustness of the association between pretreatment NLR and the cause-specific hazard of infection-related death, particularly with respect to potential bias related to adjustment for treatment variables determined after NLR assessment, we additionally performed a sensitivity analysis using an alternative IPW model restricted to three pretreatment variables: age, baseline BVAS, and eGFR. Three patients with missing data for covariates included in the propensity-score models were excluded, and complete-case analyses were performed. Covariate balance after weighting was assessed using absolute standardised mean differences, with values below 0.1 indicating adequate balance. In the weighted pseudo-population, infection-related mortality was evaluated using weighted Kaplan–Meier curves, weighted log-rank tests, and weighted Cox proportional hazards regression models, with non-infection-related deaths treated as censoring events. The proportional hazards assumption was assessed using scaled Schoenfeld residuals.

Given the exploratory nature of this study, no adjustment for multiple testing was applied to overall comparisons among the three NLR groups. Bonferroni adjustment was applied to *P* values for pairwise comparisons. All statistical analyses were performed using R software (R Foundation for Statistical Computing, Vienna, Austria) and EZR (Saitama Medical Center, Jichi Medical University, Saitama, Japan) (26). All statistical tests were two-sided, and *P* values < 0.05 were considered statistically significant.

## 3 Results

### 3.1 Baseline characteristics according to pretreatment NLR tertiles

Of the 418 patients with MPA enrolled in the REVEAL cohort, 340 newly diagnosed, treatment-naïve patients who fulfilled the eligibility criteria were included in the present study (**Supplementary Figure 2**). These patients were stratified into three groups according to pretreatment NLR tertiles: NLR1 (0.852 ≤ NLR < 4.78), NLR2 (4.78 ≤ NLR < 10.3), and NLR3 (10.3 ≤ NLR ≤ 105). Baseline characteristics are summarised in **Table 1**. Patients in the NLR3 group were significantly older than those in the other groups (*P* = 0.006), whereas sex distribution, comorbidities, and MPO-ANCA titres were comparable. The distribution of pretreatment NLR did not differ across study inclusion periods but varied among participating centres (**Supplementary Table 1**). The frequency of missing data for baseline variables did not differ significantly among the groups (**Supplementary Table 2**).

**Table 1:** Baseline characteristics of 340 patients with microscopic polyangiitis.

Variable	NLR1 (0.852 ≤ NLR < 4.78)	NLR2 (4.78 ≤ NLR < 10.3)	NLR3 (10.3 ≤ NLR ≤ 105)	*P*
N	114	113	113	
Demographics				
Age (years)	74 [67, 79]	74 [68, 80]	77 [72, 82]	0.006
Female sex (%)	66 (57.9)	64 (56.6)	65 (57.5)	0.99
BMI (kg/m^2^)	21.35 [19.89, 24.00]	21.80 [19.78, 25.00]	21.33 [19.40, 23.70]	0.66
History of smoking (%)	65 (57.5)	56 (51.9)	68 (61.8)	0.34
BI among ever-smokers	800 [350, 1150]	685 [371, 1200]	600 [340, 1030]	0.75
Coexisting diseases				
Dyslipidaemia (%)	36 (31.6)	33 (29.2)	37 (32.7)	0.85
Diabetes mellitus (%)	24 (21.1)	32 (28.3)	31 (27.4)	0.39
Hypertension (%)	60 (52.6)	51 (45.1)	61 (54.0)	0.36
Peripheral artery disease (%)	2 (1.8)	2 (1.8)	0 (0.0)	0.55
ANCA positivity				
MPO-ANCA positive (%)	114 (100)	113 (100)	113 (100)	–
MPO-ANCA titre (U/mL)	122.0 [55.3, 267.0]	181.0 [69.0, 290.0]	131.5 [56.3, 252.8]	0.1
PR3-ANCA positive (%)	4 (3.5)	6 (5.3)	2 (1.8)	0.36
Organ involvement				
DAH (%)	12 (10.5)	11 (9.7)	25 (22.1)	0.015
RPGN (%)	35 (30.7)	33 (29.2)	49 (43.4)	0.052
Interstitial lung disease (%)	70 (61.4)	56 (49.6)	41 (36.3)	0.001
UIP (%)	39 (34.2)	36 (31.9)	24 (21.2)	0.069
NSIP (%)	27 (23.7)	17 (15.0)	16 (14.2)	0.13
FFS	2.00 [2.00, 2.00]	2.00 [1.00, 2.00]	2.00 [2.00, 3.00]	0.03
BVAS	13.0 [8.0, 18.0]	15.0 [10.0, 19.0]	18.0 [13.0, 21.3]	< 0.001
Remission induction therapy				
mPSL pulse (%)	24 (21.1)	36 (31.9)	60 (53.6)	< 0.001
Initial GC dose (mg/kg)	0.85 [0.65, 0.98]	0.96 [0.66, 1.02]	0.98 [0.91, 1.04]	< 0.001
Rituximab (%)	20 (17.5)	27 (23.9)	38 (33.6)	0.021
IVCY (%)	37 (32.5)	41 (36.3)	42 (37.2)	0.72
Plasma exchange (%)	5 (4.4)	5 (4.4)	19 (16.8)	0.001
Observation period (days)	1394 [671, 2632]	1674 [540, 2518]	913 [197, 2010]	0.003

Results are expressed as median [interquartile range] for continuous variables, or the number (%) for nominal variables. ANCA, antineutrophil cytoplasmic antibody; BMI, body mass index; BI, Brinkman index; BVAS, Birmingham Vasculitis Activity Score; FFS, Five-Factor Score; GC, glucocorticoid; DAH, diffuse alveolar haemorrhage; IVCY, intravenous cyclophosphamide; MPO, myeloperoxidase; mPSL, methylprednisolone; NLR, neutrophil-to-lymphocyte ratio; NSIP, nonspecific interstitial pneumonia; PR3, proteinase 3; RPGN, rapidly progressive glomerulonephritis; UIP, usual interstitial pneumonia.

Patients with higher pretreatment NLR exhibited a distinct clinical phenotype. DAH was significantly more frequent in the NLR3 group (22.1%) than in the NLR1 (10.5%) and NLR2 (9.7%) groups (*P* = 0.015), whereas rapidly progressive glomerulonephritis (RPGN) also tended to be more common in the NLR3 group (*P* = 0.052). In contrast, ILD was significantly more prevalent in the NLR1 group (61.4%, *P* = 0.001). Although usual interstitial pneumonia (UIP) and non-specific interstitial pneumonia (NSIP) patterns both tended to occur more frequently in the NLR1 group, neither association reached statistical significance.

Higher pretreatment NLR was also associated with greater disease severity and disease activity. Patients in the NLR3 group had significantly higher FFS and total BVAS, together with higher general, chest, and renal BVAS subscores (**Supplementary Table 3**). Pretreatment NLR showed a modest positive correlation with BVAS (*r* = 0.257, 95% CI 0.16–0.35, **Supplementary Figure 3**). Consistent with these findings, patients in the NLR3 group more frequently received methylprednisolone pulse therapy, higher initial GC doses, rituximab, and plasma exchange, whereas intravenous cyclophosphamide use was similar among the groups (**Table 1**). The use of avacopan during the treatment course was comparable among the groups (**Supplementary Table 4**).

### 3.2 Laboratory findings according to pretreatment NLR tertiles

The laboratory findings are summarised in **Table 2**. As expected, the NLR3 group had higher neutrophil counts and lower lymphocyte counts, resulting in higher NLR values across the tertiles. Patients in the NLR3 group also exhibited laboratory findings consistent with more active systemic inflammation, including lower haemoglobin and serum albumin levels and higher platelet counts and CRP concentrations (all *P* < 0.001). Consistent with the differences in organ involvement, eGFR was significantly lower in the NLR3 group (*P* = 0.003), whereas serum KL-6 concentrations were highest in the NLR1 group (*P* < 0.001). Serum IgG concentrations were significantly lower in the NLR3 group (*P* < 0.001).

**Table 2:** Comparison of laboratory findings at baseline.

Variable	NLR1 (0.852 ≤ NLR < 4.78)	NLR2 (4.78 ≤ NLR < 10.3)	NLR3 (10.3 ≤ NLR ≤ 105)	*P*
White blood cell (/μL)	7885 [6233, 9935]	11390 [9200, 13580]	13800 [11810, 16690]	< 0.001
Neutrophils (/μL)	5236 [3993, 6515]	8717 [7227, 10820]	12240 [10168, 14353]	< 0.001
Lymphocytes (/μL)	1736 [1389, 2128]	1260 [974, 1630]	772 [599, 966]	< 0.001
NLR	3.22 [2.42, 3.92]	6.99 [5.70, 8.58]	14.66 [12.32, 19.39]	–
Haemoglobin (g/dL)	10.65 [8.93, 12.30]	10.00 [8.80, 11.40]	9.40 [8.30, 10.60]	< 0.001
Platelet (×10^4^/μL)	29.50 [23.02, 36.55]	39.30 [30.60, 49.10]	39.70 [29.10, 47.40]	< 0.001
Albumin (g/dL)	3.30 [2.70, 3.60]	2.50 [2.10, 2.80]	2.30 [1.90, 2.60]	< 0.001
LDH (U/L)	189.5 [165.5, 229.0]	188.0 [156.0, 215.0]	187.0 [154.0, 236.0]	0.67
KL-6 (U/mL)	403.5 [248.3, 764.8]	258.5 [176.5, 476.7]	236.5 [154.3, 324.0]	< 0.001
eGFR (mL/min/1.73m^2^)	49.85 [30.13, 76.15]	49.90 [26.30, 73.77]	30.84 [14.90, 62.71]	0.003
CRP (mg/dL)	2.47 [0.51, 7.12]	9.83 [6.60, 13.50]	12.28 [9.08, 16.13]	< 0.001
Serum IgG (mg/dL)	1690 [1413, 2072]	1672 [1409, 2078]	1407 [1208, 1665]	< 0.001
Microscopic haematuria (%)	76 (74.5)	72 (71.3)	83 (77.6)	0.60

Results are expressed as median [interquartile range] for continuous variables, or the number (%) for nominal variables. ANCA, antineutrophil cytoplasmic antibody; CRP, C-reactive protein; eGFR, estimated glomerular filtration rate; LDH, lactate dehydrogenase; NLR, neutrophil-to-lymphocyte ratio.

### 3.3 Disease course according to pretreatment NLR tertiles

Although patients in the NLR3 group had significantly higher BVAS and received higher initial GC doses at disease onset, these between-group differences disappeared after treatment initiation, possibly reflecting regression to the mean (**Supplementary Figure 4**). Likewise, VDI values were comparable among the three groups (**Supplementary Figure 5**). The frequency of missing longitudinal data for GC dose, BVAS, and VDI was higher in the NLR3 group, reflecting the greater number of early deaths in this group (**Supplementary Table 5**).

Because early mortality might have influenced the analyses, a sensitivity analysis restricted to patients who completed 12 months of follow-up was performed. Similar findings were observed, with no significant differences in BVAS or GC dose after treatment initiation (**Supplementary Figure 6**).

### 3.4 Survival analyses

Relapse-free survival did not differ significantly among the three NLR groups over the 5-year follow-up period (*P* = 0.44). Similarly, neither major-relapse-free survival (*P* = 0.80) nor minor-relapse-free survival (*P* = 0.57) differed significantly among the groups (**Figures 1A–C**).

**Figure 1:**
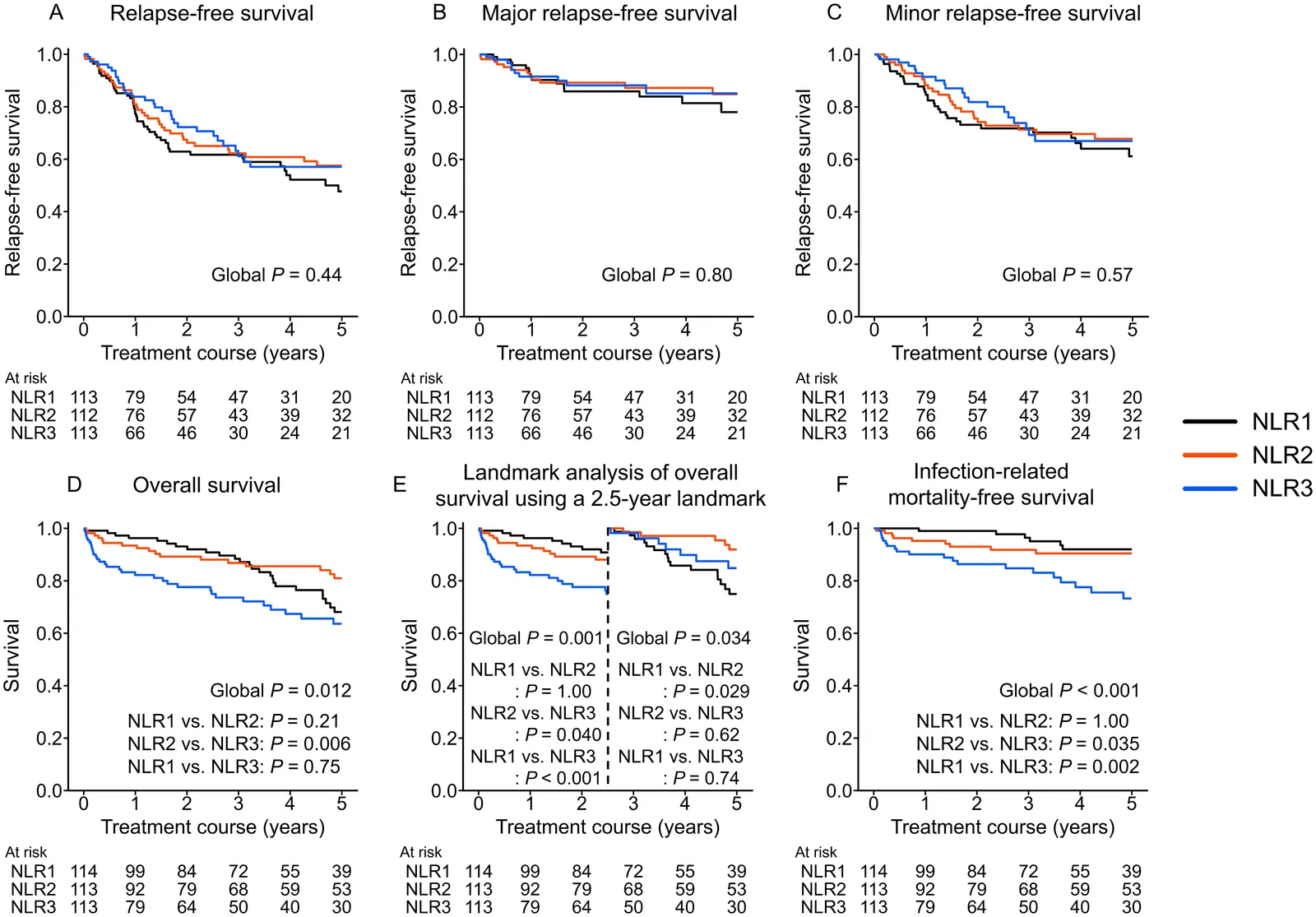
Kaplan–Meier analyses according to pretreatment NLR tertiles. **(A)** Relapse-free survival. **(B)** Major relapse-free survival. **(C)** Minor relapse-free survival. **(D)** Overall survival. **(E)** Landmark analysis of overall survival using a 2.5-year landmark. **(F)** Infection-related mortality-free survival. Survival curves were estimated using the Kaplan–Meier method and compared using the log-rank test. Pairwise comparisons were adjusted using the Bonferroni correction where applicable. NLR, neutrophil-to-lymphocyte ratio.

In contrast, overall survival differed significantly according to pretreatment NLR (*P* = 0.012), with the NLR3 group showing the poorest prognosis (**Figure 1D**). Because the Kaplan–Meier survival curves crossed during follow-up, suggesting potential non-proportional hazards, landmark analyses were subsequently performed using a landmark at 2.5 years. Before the landmark, overall survival differed significantly among the groups (*P* = 0.001), with significantly poorer survival in the NLR3 group than in both the NLR1 (*P* < 0.001) and NLR2 (*P* = 0.040) groups. In contrast, after the landmark, survival remained significantly different (*P* = 0.034), primarily because the NLR1 group exhibited poorer late survival than the NLR2 group (*P* = 0.029), whereas no significant differences were observed between the NLR3 group and either of the other groups (**Figure 1E**). Sensitivity analyses using 2-year and 3-year landmark time points yielded similar findings (**Supplementary Figure 7**).

### 3.5 Cause-specific mortality and inverse probability weighting analyses

To better understand the difference in overall survival, we next examined cause-specific mortality (**Table 3**). Among the predefined causes of death, infection-related mortality was the only outcome that differed significantly among the NLR groups. Although ILD and DAH were more prevalent at baseline in different NLR groups, neither ILD-related mortality nor DAH-related mortality differed significantly during follow-up. In contrast, infection-related mortality was significantly more frequent in the NLR3 group (*P* = 0.002), and infection-related mortality-free survival was significantly lower in the NLR3 group than in the NLR1 (*P* = 0.002) and NLR2 (*P* = 0.035) groups, as assessed using pairwise log-rank tests (**Figure 1F**). Similarly, in the competing-risk analysis treating non-infection-related deaths as competing events, the cumulative incidence of infection-related death was significantly higher in the NLR3 group than in the NLR1 group (*P* = 0.004) and tended to be higher than in the NLR2 group (*P* = 0.057) (**Figure 2**). When NLR was analysed as a continuous variable, its overall association with infection-related mortality remained statistically significant (*P* = 0.042), and the estimated HR generally increased with higher NLR (**Supplementary Figure 8**). However, the pointwise 95% confidence intervals were wide and included 1 over a wide range of NLR values. There was no evidence of non-linearity in the association between log10-transformed NLR and infection-related mortality. Martingale residuals showed no evident systematic pattern, and tests based on scaled Schoenfeld residuals provided no evidence of violation of the proportional hazards assumption (**Supplementary Figure 9**).

**Table 3:** Cause-specific mortality incidence rate ratios.

	Crude death rate (/100 patient-years)			The exact Poisson test	
Variables	NLR1 (0.852 ≤ NLR < 4.78)	NLR2 (4.78 ≤ NLR < 10.3)	NLR3 (10.3 ≤ NLR ≤ 105)	NLR3 vs. NLR1	
				IRR [95% CI]	*P*
All-cause mortality	5.81 (33)	5.26 (28)	8.29 (33)	1.43 [0.85, 2.39]	0.17
Acute exacerbation of ILD	0.88 (5)	0.56 (3)	0.00 (0)	0 [0, 1.56]	0.082
DAH	0.35 (2)	0.19 (1)	1.26 (5)	3.57 [0.58, 37.5]	0.13
Gastrointestinal bleeding/perforation	0.18 (1)	0.19 (1)	0.75 (3)	4.28 [0.34, 225]	0.31
Renal failure	0.18 (1)	0.00 (0)	0.00 (0)	0 [0, 55.7]	1.00
Infections	1.58 (9)	2.07 (11)	5.28 (21)	3.33 [1.46, 8.26]	0.002
Others	1.76 (10)	2.07 (11)	0.75 (3)	0.43 [0.08, 1.66]	0.26
Unknown	0.88 (5)	0.19 (1)	0.25 (1)	0.29 [0.01, 2.55]	0.41

Values are presented as crude death rates per 100 patient-years (number of deaths).

DAH, diffuse alveolar haemorrhage; ILD, interstitial lung disease; IRR, incidence rate ratio; NLR, neutrophil-to-lymphocyte ratio.

**Figure 2:**
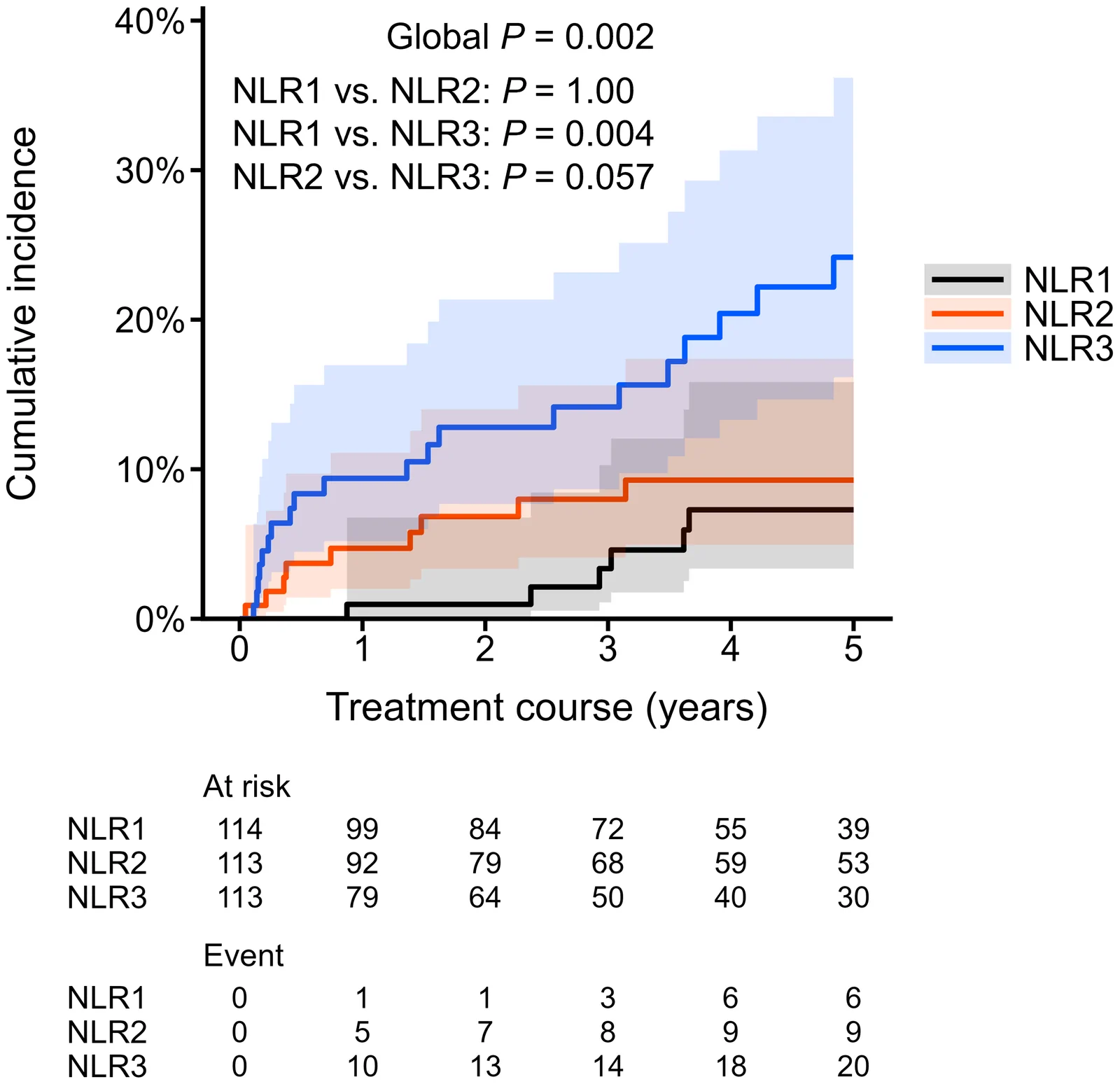
Cumulative incidence of infection-related death according to pretreatment NLR tertiles. Cumulative incidence curves for infection-related death are shown, with non-infection-related deaths treated as competing events. Shaded areas indicate 95% confidence intervals. Differences among the NLR groups were assessed using Gray’s test. Pairwise comparisons were adjusted using the Bonferroni correction where applicable. NLR, neutrophil-to-lymphocyte ratio.

The specific causes and numbers of infection-related deaths are shown in **Supplementary Figure 10**. The number of deaths due to pneumonia was similar across the NLR groups. Deaths due to sepsis of unknown source were more frequent in the NLR3 group. Opportunistic infections, including Pneumocystis pneumonia (PCP), cytomegalovirus pneumonia, and invasive pulmonary aspergillosis, accounted for 1, 1, and 3 deaths in the NLR1, NLR2, and NLR3 groups, respectively. Among these opportunistic infections, PCP occurred only in the NLR3 group. Because patients in the NLR3 group were older, had higher disease activity, and received more intensive remission-induction therapy, it remained unclear whether the increased risk of infection-related mortality reflected baseline disease severity or was independently associated with pretreatment NLR. Therefore, stabilised inverse probability weighting was performed to account for measured differences among the NLR groups. After weighting, covariate balance was achieved, with absolute standardised mean differences below 0.1 for all adjustment variables (**Supplementary Figure 11**), although ILD remained more prevalent in the NLR1 group (**Supplementary Table 6**).

After IPW adjustment, overall survival no longer differed significantly among the three groups (**Figure 3A**). In contrast, infection-related mortality-free survival continued to differ significantly among the NLR groups in the global weighted log-rank test (**Figure 3B**). The NLR3 group showed the lowest infection-related mortality-free survival, although none of the pairwise comparisons reached statistical significance. In the weighted Cox proportional hazards analysis, the NLR3 group had a significantly higher hazard of infection-related death than the NLR1 group (HR 3.47, 95% CI 1.32–9.09, *P* = 0.034), whereas no significant difference was observed between the NLR2 and NLR3 groups (**Supplementary Table 7**). In the sensitivity analysis using an alternative IPW model restricted to pretreatment variables, the global weighted log-rank test indicated a significant difference in infection-related mortality-free survival among the NLR groups (*P* = 0.048). The NLR3 group showed the lowest infection-related mortality-free survival, but, as in the primary analysis, none of the pairwise comparisons reached statistical significance after Bonferroni correction (**Supplementary Figure 12**). Similarly, the weighted Cox proportional hazards model yielded a higher hazard of infection-related death in the NLR3 group than in the NLR1 group, consistent with the direction of the primary analysis, although this comparison was not statistically significant after Bonferroni correction (HR 2.90, nominal 95% CI 1.16–7.26, nominal *P* = 0.023, Bonferroni-adjusted *P* = 0.068) (**Supplementary Table 8**). The distributions of pretreatment covariates after weighting and residual diagnostics for the weighted Cox models used in the primary and sensitivity analyses are presented in the **Supplementary Materials** (**Supplementary Table 9**; **Supplementary Figure 13**, **14**).

**Figure 3:**
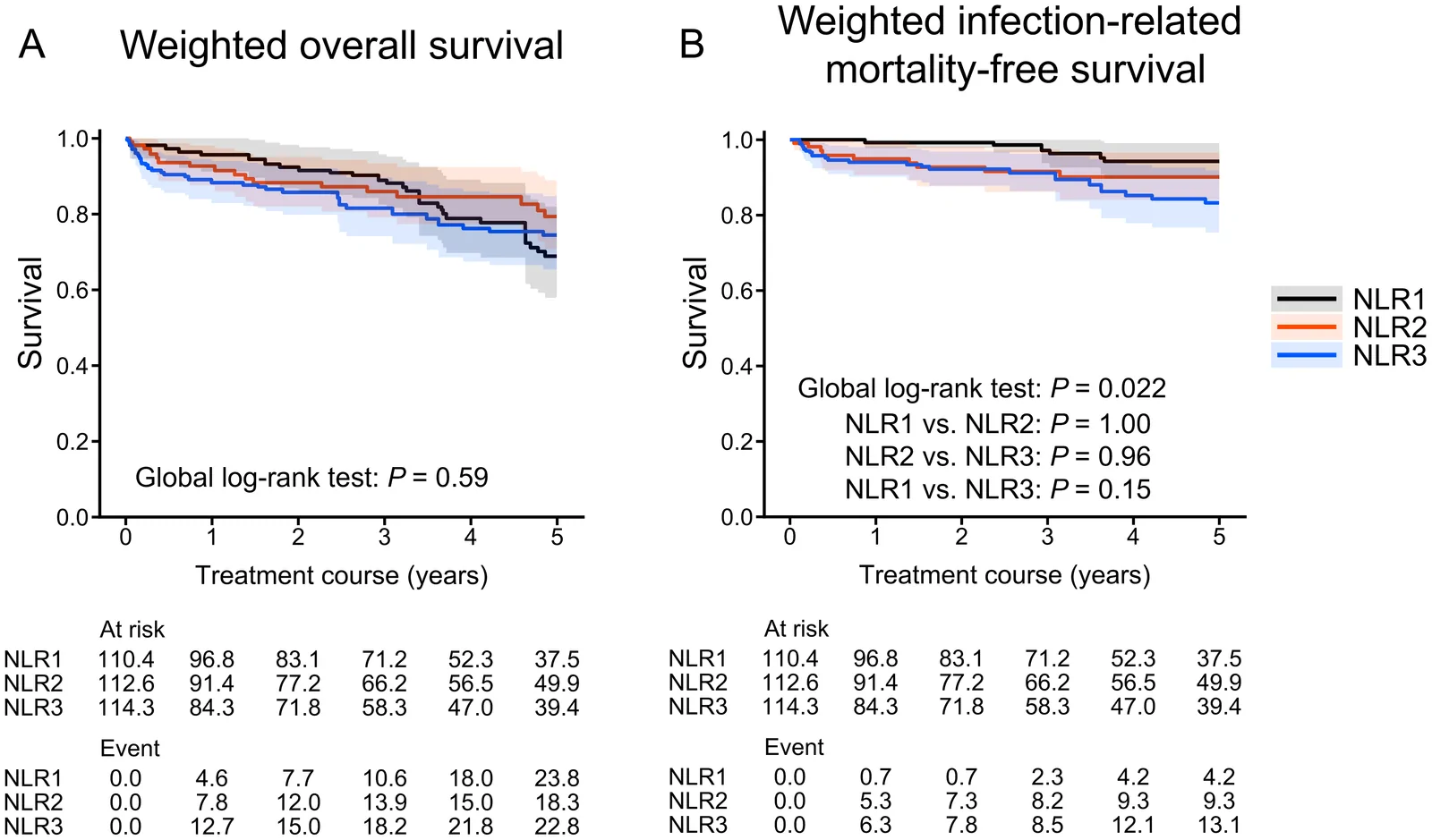
Inverse probability weighting analyses of survival outcomes. **(A)** Weighted overall survival. **(B)** Weighted infection-related mortality-free survival. Survival curves were estimated after stabilised inverse probability weighting to balance baseline characteristics among the three NLR groups. Shaded areas indicate 95% confidence intervals. Differences among the groups were assessed using weighted log-rank tests. Pairwise comparisons were adjusted using the Bonferroni correction where applicable. The tables below the curves show weighted numbers at risk and weighted cumulative event counts. NLR, neutrophil-to-lymphocyte ratio.

## 4 Discussion

In this multicentre cohort study of patients with newly diagnosed MPA, we demonstrated that pretreatment NLR was associated with greater disease severity at presentation, distinct clinical phenotypes, and poorer long-term survival. Although patients with higher pretreatment NLR required more intensive remission-induction therapy, subsequent disease activity, GC tapering, and cumulative organ damage became comparable among the three groups. Most importantly, pretreatment NLR remained independently associated with infection-related mortality after adjustment for baseline disease severity and treatment intensity using IPW. These findings suggest that pretreatment NLR is not simply a marker of systemic inflammation but may represent a practical biomarker for early risk stratification in patients with MPA.

One of the most novel observations of this study was the association between pretreatment NLR and distinct clinical phenotypes of MPA. Patients with higher pretreatment NLR more frequently presented with DAH, impaired renal function, and a tendency toward RPGN, whereas ILD was substantially more common among patients with lower NLR. Previous studies in non-AAV populations have generally reported that elevated NLR is associated with the presence of ILD and poorer outcomes in patients with fibrotic lung disease (27, 28). At first glance, these observations appear inconsistent with our findings. However, this discrepancy is likely explained by the unique clinical phenotype of MPO-ANCA-associated ILD. Unlike idiopathic fibrotic lung diseases, ILD in MPO-ANCA-associated AAV often precedes the onset of systemic vasculitis and has been associated with relatively mild vasculitic activity and lower systemic inflammatory burden (29–31). Consequently, patients with ILD may exhibit lower circulating NLR despite substantial pulmonary fibrosis. In contrast, DAH and RPGN are characterised by excessive neutrophil activation and neutrophil extracellular trap formation, providing a plausible biological explanation for the markedly elevated pretreatment NLR observed in these patients. Collectively, these findings suggest that pretreatment NLR may reflect not merely the magnitude of systemic inflammation but also distinct inflammatory phenotypes within MPA.

Consistent with previous studies (5, 32), pretreatment NLR was positively associated with disease severity, as reflected by higher BVAS and FFS, and patients with higher pretreatment NLR consequently received more intensive remission-induction therapy. Despite these marked differences at presentation, the disease activity, GC tapering, and cumulative organ damage became comparable among the three groups following treatment initiation. These findings suggest that pretreatment NLR primarily reflects inflammatory burden at disease onset rather than resistance to immunosuppressive therapy.

Interestingly, although a previous study restricted to MPO-ANCA-associated AAV reported no association between baseline NLR and renal outcome (33), patients with higher pretreatment NLR in our cohort had poorer renal function and a tendency toward RPGN, suggesting substantial heterogeneity even within MPO-ANCA-associated AAV.

The most clinically important finding of this study was that pretreatment NLR was independently associated with infection-related mortality. Several previous studies have reported that elevated NLR is related to all-cause mortality and infection-related mortality in AAV (5, 15, 32, 33). However, these studies were limited by substantial heterogeneity in baseline characteristics and treatment exposure, including established risk factors for infection-related mortality such as older age, impaired renal function, high disease activity, and intensive immunosuppressive therapy (34, 35). Our findings extend the existing literature by demonstrating that the association between pretreatment NLR and infection-related mortality persisted even after rigorous adjustment for baseline disease severity and treatment intensity using IPW. In contrast, the difference in overall survival was attenuated after adjustment, suggesting that pretreatment NLR may be more closely associated with an increased risk of fatal infection than with poor prognosis in general.

Interestingly, relapse-free survival did not differ among the NLR groups, in contrast to previous studies of mixed AAV cohorts (32, 36). This discrepancy may reflect the restriction of the present study to patients with MPA, which is characterised by a lower relapse rate and different disease biology compared with PR3-ANCA-positive disease.

The observed association between elevated pretreatment NLR and infection-related mortality is biologically plausible. Elevated NLR has been associated with postoperative infectious complications even in patients without autoimmune disease or immunosuppressive therapy (37, 38), suggesting that NLR reflects an intrinsic vulnerability to infection rather than merely treatment-related immunosuppression. From a clinical perspective, patients with markedly elevated pretreatment NLR may therefore benefit from intensified infection surveillance, optimisation of GC exposure, and appropriate infection prophylaxis during remission induction.

The mechanisms linking elevated pretreatment NLR to infection-related mortality remain incompletely understood. NLR reflects the balance between innate and adaptive immunity by integrating neutrophilia driven by systemic inflammation and relative lymphopenia, which may indicate impaired adaptive immune competence. Previous studies have shown that excessive neutrophil activation contributes directly to endothelial injury, neutrophil extracellular trap formation, and tissue damage in AAV, whereas lymphopenia has consistently been associated with increased susceptibility to severe infections in autoimmune diseases (2, 39, 40). Accordingly, an elevated pretreatment NLR may reflect a state of heightened inflammatory activity accompanied by reduced immunological reserve, potentially increasing susceptibility to fatal infectious complications following immunosuppressive therapy. The lower baseline serum IgG concentrations observed in the highest NLR group may further support the concept of reduced immunological reserve in these patients. Future studies integrating immune-cell phenotyping and functional analyses are warranted to clarify these mechanisms.

This study has several strengths. To our knowledge, it is the largest multicentre study specifically evaluating the clinical significance of pretreatment NLR exclusively in patients with newly diagnosed MPA. Restricting the study population to treatment-naïve patients minimised the influence of previous immunosuppressive therapy on peripheral blood cell counts. In addition, the combination of landmark analyses and IPW-adjusted survival analyses enabled comprehensive evaluation of long-term prognosis. Finally, the multicentre REVEAL cohort reflects routine clinical practice across multiple tertiary referral centres, enhancing the robustness and clinical relevance of our findings. These methodological strengths increase the robustness of the observed association between pretreatment NLR and infection-related mortality.

Several limitations should be acknowledged. First, this was a retrospective observational study, and residual confounding cannot be completely excluded despite extensive adjustment using IPW. Second, treatment strategies were determined by the treating physicians rather than by a standardised protocol, introducing potential treatment-selection bias. Third, because the study population consisted exclusively of Japanese patients with predominantly MPO-ANCA-positive MPA, the generalisability of our findings to other populations remains uncertain. Fourth, higher early mortality in the NLR3 group resulted in shorter follow-up and may have introduced survivorship bias into longitudinal analyses at later time points. In our study, between-group differences in GC dose and BVAS were no longer evident after treatment initiation, possibly partly reflecting regression to the mean. Similar findings were obtained in the sensitivity analysis restricted to patients who completed 12 months of follow-up. Nevertheless, patients who died early did not contribute to subsequent follow-up assessments, and survivorship bias cannot be excluded. Accordingly, longitudinal findings at later time points should be interpreted with caution. Fifth, although patients receiving any GC therapy at baseline were excluded and their GC dose at pretreatment blood sampling was confirmed to be 0 mg, prior GC exposure before referral could not be completely excluded because data were collected retrospectively across multiple centres over a 20-year period. Sixth, because the distribution of pretreatment NLR varied among participating centres, some of our findings may have been influenced by differences in their roles within regional healthcare systems and centre-specific treatment practices. Seventh, serum albumin was not included in the propensity-score model because of limited covariate overlap across the NLR groups, particularly between the NLR1 and NLR3 groups, which resulted in disproportionately large weights for a small number of patients and a substantial reduction in the effective sample size. Consequently, residual confounding related to serum albumin cannot be excluded. Finally, detailed information regarding infection prophylaxis, including trimethoprim-sulfamethoxazole use and vaccination status, was unavailable, although these factors may have influenced infection-related outcomes.

## 5 Conclusion

In conclusion, pretreatment NLR was associated with greater disease severity, distinct clinical phenotypes, and infection-related mortality in patients with newly diagnosed MPA in Japan. NLR is an inexpensive and universally available biomarker that can be readily calculated from routine blood tests. Therefore, whether NLR can be used to identify patients who may benefit from enhanced infection-prevention strategies is an important clinical question. Further studies in diverse populations are warranted to validate these findings and clarify the potential role of NLR in guiding infection prevention.

## Data Availability

The original contributions presented in the study are included in the article/**Supplementary Material**. Further inquiries can be directed to the corresponding author.
