# Supplementary material for: Pretreatment neutrophil-to-lymphocyte ratio is associated with clinical phenotypes and infection-related mortality in microscopic polyangiitis: the multicentre REVEAL cohort study
Source: Front Immunol. 2026 Oct 19;17:1948318. doi: 10.3389/fimmu.2026.1948318 (PMC13724462; doi:10.3389/fimmu.2026.1948318)
Supplement: Supplementary file 1 [file SupplementaryFile1.docx]

Supplementary Material

# 1 Supplementary Tables

**Supplementary Table 1. Pretreatment neutrophil-to-lymphocyte ratio by participating centre and study inclusion period**

| **Variable** | **N** | **Pretreatment NLR** | ***P*** |
| --- | --- | --- | --- |
| <Study inclusion period> |  |  | 0.31 |
| 2005–2009 | 15 | 7.51 [3.74, 12.75] |  |
| 2010–2014 | 55 | 5.18 [3.24, 12.56] |  |
| 2015–2019 | 111 | 6.27 [3.98, 10.94] |  |
| 2020–2025 | 159 | 7.99 [4.20, 13.84] |  |
| <Participating centre> |  |  | 0.005 |
| KCHO | 59 | 9.16 [4.52, 13.25] |  |
| KUHP | 54 | 6.65 [4.61, 13.99] |  |
| OMPU | 138 | 6.21 [3.94, 12.13] |  |
| OMU | 24 | 5.72 [2.91, 7.87] |  |
| TYZ-P | 35 | 8.42 [4.98, 13.23] |  |
| YCH | 30 | 3.51 [2.52, 9.79] |  |

Results are expressed as median [interquartile range]. KCHO, Kobe City Medical Center General Hospital; KUHP, Kyoto University Hospital; NLR, neutrophil-to-lymphocyte ratio; OMPU, Osaka Medical and Pharmaceutical University; OMU, Osaka Metropolitan University; TYZ-P, Tenri Hospital; YCH, Yodogawa Christian Hospital.

**Supplementary Table 2. Frequency of missing data for baseline characteristics and treatment variables**

| **Variable** | **NLR1**  **(0.852 ≤ NLR < 4.78)** | **NLR2**  **(4.78 ≤ NLR < 10.3)** | **NLR3**  **(10.3 ≤ NLR ≤ 105)** | ***P*** |
| --- | --- | --- | --- | --- |
| N | 114 | 113 | 113 |  |
| Sex | 0 | 0 | 0 | 1.0 |
| Age | 0 | 0 | 0 | 1.0 |
| BMI | 2 | 0 | 0 | 0.33 |
| History of smoking | 1 | 5 | 3 | 0.21 |
| Dyslipidaemia | 0 | 0 | 0 | 1.0 |
| Diabetes mellitus | 0 | 0 | 0 | 1.0 |
| Hypertension | 0 | 0 | 0 | 1.0 |
| Peripheral artery disease | 0 | 0 | 1 | 0.67 |
| DAH | 0 | 0 | 0 | 1.0 |
| RPGN | 0 | 0 | 0 | 1.0 |
| ILD | 0 | 0 | 0 | 1.0 |
| FFS | 0 | 0 | 0 | 1.0 |
| BVAS | 0 | 0 | 1 | 0.67 |
| mPSL pulse | 0 | 0 | 1 | 0.67 |
| Initial GC dose | 0 | 0 | 0 | 1.0 |
| Rituximab | 0 | 0 | 0 | 1.0 |
| IVCY | 0 | 0 | 0 | 1.0 |
| Plasma exchange | 0 | 0 | 0 | 1.0 |
| White blood cell | 0 | 0 | 0 | 1.0 |
| Neutrophils | 0 | 0 | 0 | 1.0 |
| Lymphocytes | 0 | 0 | 0 | 1.0 |
| NLR | 0 | 0 | 0 | 1.0 |
| Haemoglobin | 0 | 0 | 0 | 1.0 |
| Platelet | 0 | 0 | 0 | 1.0 |
| Albumin | 0 | 1 | 0 | 0.67 |
| LDH | 0 | 0 | 0 | 1.0 |
| KL-6 | 14 | 23 | 23 | 0.17 |
| eGFR | 0 | 0 | 0 | 1.0 |
| CRP | 0 | 0 | 0 | 1.0 |
| Serum IgG | 19 | 19 | 11 | 0.23 |
| Microscopic haematuria | 12 | 12 | 6 | 0.29 |

Values indicate the numbers of patients with missing data. BMI, body mass index; BVAS, Birmingham Vasculitis Activity Score; CRP, C-reactive protein; DAH, diffuse alveolar haemorrhage; eGFR, estimated glomerular filtration rate; FFS, Five-Factor Score; GC, glucocorticoid; IgG, immunoglobulin G; ILD, interstitial lung disease; IVCY, intravenous cyclophosphamide; LDH, lactate dehydrogenase; mPSL, methylprednisolone; NLR, neutrophil-to-lymphocyte ratio; RPGN, rapidly progressive glomerulonephritis.

**Supplementary Table 3. The Birmingham Vasculitis Activity Score by organ system at disease onset**

| **Variable** | **NLR1**  **(0.852 ≤ NLR < 4.78)** | **NLR2**  **(4.78 ≤ NLR < 10.3)** | **NLR3**  **(10.3 ≤ NLR ≤ 105)** | ***P*** |
| --- | --- | --- | --- | --- |
| Total score  at the onset | 13.0 [8.0, 18.0] | 15.0 [10.0, 19.0] | 18.0 [13.0, 21.3] | < 0.001 |
| General | 1.0 [0, 2.0] | 2.0 [1.0, 3.0] | 2.0 [1.0, 3.0] | < 0.001 |
| Cutaneous | 0 [0, 0] | 0 [0, 0] | 0 [0, 0] | 0.85 |
| Mucous membranes/eyes | 0 [0, 0] | 0 [0, 0] | 0 [0, 0] | 0.034 |
| Ear, nose, and throat | 0 [0, 0] | 0 [0, 0] | 0 [0, 0] | 0.52 |
| Chest | 0 [0, 4.0] | 0 [0, 4.0] | 3.0 [0, 6.0] | 0.001 |
| Cardiovascular | 0 [0, 0] | 0 [0, 0] | 0 [0, 0] | 0.16 |
| Abdominal | 0 [0, 0] | 0 [0, 0] | 0 [0, 0] | 0.37 |
| Renal | 6.0 [0, 12.0] | 10.0 [0, 12.0] | 10.0 [6.0, 12.0] | 0.022 |
| Nervous system | 0 [0, 6.0] | 0 [0, 6.0] | 0 [0, 6.0] | 0.53 |

Results are expressed as median [interquartile range]. NLR, neutrophil-to-lymphocyte ratio.

**Supplementary Table 4. The use of avacopan during the treatment course**

| **Variable** | **NLR1**  **(0.852 ≤ NLR < 4.78)** | **NLR2**  **(4.78 ≤ NLR < 10.3)** | **NLR3**  **(10.3 ≤ NLR ≤ 105)** | ***P*** |
| --- | --- | --- | --- | --- |
| Avacopan | 21 (18.4) | 19 (16.8) | 17 (15.0) | 0.79 |

Results are expressed as the number (%). NLR, neutrophil-to-lymphocyte ratio.

**Supplementary Table 5. Frequency of missing data in longitudinal variables**

| **Variable** | **NLR1**  **(0.852 ≤ NLR < 4.78)** | **NLR2**  **(4.78 ≤ NLR < 10.3)** | **NLR3**  **(10.3 ≤ NLR ≤ 105)** | ***P*** |
| --- | --- | --- | --- | --- |
| N | 114 | 113 | 113 |  |
| GC dose at 3 months | 7 | 9 | 16 | 0.10 |
| GC dose at 6 months | 11 | 15 | 26 | 0.017 |
| GC dose at 12 months | 16 | 20 | 34 | 0.009 |
| GC dose at 24 months | 31 | 33 | 47 | 0.047 |
| BVAS at 6 months | 10 | 16 | 30 | 0.001 |
| BVAS at 12 months | 16 | 20 | 37 | 0.002 |
| BVAS at 24 months | 30 | 33 | 49 | 0.015 |
| VDI at 6 months | 10 | 16 | 30 | 0.001 |
| VDI at 12 months | 16 | 20 | 37 | 0.002 |
| VDI at 24 months | 30 | 33 | 49 | 0.015 |

BVAS, Birmingham Vasculitis Activity Score; GC, glucocorticoid; NLR, neutrophil-to-lymphocyte ratio; VDI, Vasculitis Damage Index.

**Supplementary Table 6. Clinical characteristics after stabilised inverse probability weighting**

| **Variable** | **NLR1**  **(0.852 ≤ NLR < 4.78)** | **NLR2**  **(4.78 ≤ NLR < 10.3)** | **NLR3**  **(10.3 ≤ NLR ≤ 105)** | ***P*** |
| --- | --- | --- | --- | --- |
| N | 110.4 | 112.6 | 114.3 | - |
| Age (years) | 73.3 (9.2) | 73.9 (9.8) | 73.1 (14.7) | 0.86 |
| BVAS | 14.2 (6.9) | 14.7 (7.7) | 14.6 (7.0) | 0.89 |
| Interstitial lung disease (%) | 66.1 (59.8) | 54.3 (48.2) | 38.7 (33.8) | 0.002 |
| DAH (%) | 16.5 (15.0) | 13.1 (11.6) | 19.1 (16.7) | 0.66 |
| RPGN (%) | 41.3 (37.4) | 35.1 (31.2) | 36.2 (31.6) | 0.601 |
| eGFR (mL/min/1.73m^2^) | 46.3 (31.4) | 50.4 (29.8) | 55.5 (45.8) | 0.40 |
| mPSL pulse (%) | 39.0 (35.3) | 39.2 (34.8) | 40.5 (35.4) | 1.00 |
| Initial GC dose (mg/kg) | 0.88 (0.23) | 0.87 (0.22) | 0.88 (0.22) | 1.00 |
| Rituximab (%) | 25.7 (23.2) | 29.1 (25.8) | 27.7 (24.3) | 0.92 |
| IVCY (%) | 40.9 (37.0) | 41.0 (36.4) | 40.9 (35.8) | 0.98 |

Results are expressed as weighted mean (standard deviation) for continuous variables and weighted number (%) for categorical variables. BVAS, Birmingham Vasculitis Activity Score; DAH, diffuse alveolar haemorrhage; eGFR, estimated glomerular filtration rate; GC, glucocorticoid; mPSL, methylprednisolone; IVCY, intravenous cyclophosphamide; NLR, neutrophil-to-lymphocyte ratio; RPGN, rapidly progressive glomerulonephritis.

**Supplementary Table 7. Weighted Cox proportional hazards analysis for infection-related mortality**

| **Variable** | **Reference** | **Hazard ratio [95% CI]** | ***P*** |
| --- | --- | --- | --- |
| NLR3 | NLR1 | 3.47 [1.32, 9.09] | 0.034 |
|  | NLR2 | 1.52 [0.66, 3.51] | 0.98 |
| NLR2 | NLR1 | 2.28 [0.79, 6.61] | 0.39 |

*P* values were adjusted using the Bonferroni method. Confidence intervals are nominal 95% confidence intervals. NLR, neutrophil-to-lymphocyte ratio.

**Supplementary Table 8. Sensitivity analysis of infection-related mortality using a weighted Cox proportional hazards model**

| **Variable** | **Reference** | **Hazard ratio [95% CI]** | ***P*** |
| --- | --- | --- | --- |
| NLR3 | NLR1 | 2.90 [1.16, 7.26] | 0.068 |
|  | NLR2 | 1.78 [0.77, 4.11] | 0.53 |
| NLR2 | NLR1 | 1.63 [0.57, 4.69] | 1.00 |

*P* values were adjusted using the Bonferroni method. Confidence intervals are nominal 95% confidence intervals. NLR, neutrophil-to-lymphocyte ratio.

**Supplementary Table 9. Distribution of pretreatment covariates after stabilised inverse probability weighting in the sensitivity analysis**

| **Variable** | **NLR1**  **(0.852 ≤ NLR < 4.78)** | **NLR2**  **(4.78 ≤ NLR < 10.3)** | **NLR3**  **(10.3 ≤ NLR ≤ 105)** | ***P*** |
| --- | --- | --- | --- | --- |
| N | 111.0 | 113.8 | 110.0 | - |
| Age (years) | 73.7 (9.9) | 74.0 (10.1) | 74.5 (9.7) | 0.83 |
| BVAS | 14.4 (7.1) | 14.6 (7.2) | 14.8 (7.0) | 0.91 |
| eGFR (mL/min/1.73m^2^) | 48.2 (29.7) | 48.8 (29.3) | 49.7 (41.9) | 0.96 |

Results are expressed as weighted mean (standard deviation).

BVAS, Birmingham Vasculitis Activity Score; eGFR, estimated glomerular filtration rate; NLR, neutrophil-to-lymphocyte ratio.

# 2 Supplementary Figures

**Supplementary Figure 1.** **Directed acyclic graph illustrating the hypothesised relationships among pretreatment NLR, disease activity, treatment intensity, and infection-related mortality**

Underlying disease activity was assumed to influence pretreatment NLR, disease activity captured by BVAS, treatment intensity, and infection-related mortality. Disease activity captured by BVAS and treatment intensity were considered to represent separate pathways linking underlying disease activity to infection-related mortality. The outer lower pathway represents a hypothesised residual pathway involving disease activity that is neither captured by BVAS nor reflected in treatment intensity. Age and baseline eGFR were considered pretreatment covariates influencing both pretreatment NLR and infection-related mortality. Treatment intensity represents the initial glucocorticoid dose, methylprednisolone pulse therapy, rituximab, and intravenous cyclophosphamide. BVAS, Birmingham Vasculitis Activity Score; eGFR, estimated glomerular filtration rate; NLR, neutrophil-to-lymphocyte ratio.

**Supplementary Figure 2. Study flowchart**

Flowchart illustrating patient selection from the multicentre REVEAL cohort. Of 418 MPA patients, 340 patients were included. Patients were stratified into tertiles according to pretreatment NLR.

ACR/EULAR, American College of Rheumatology/European Alliance of Associations for Rheumatology; CHCC2012, Chapel Hill Consensus Conference 2012; MPA, microscopic polyangiitis; NLR, neutrophil-to-lymphocyte ratio.

**Supplementary Figure 3. Correlation between pretreatment NLR and baseline BVAS**

Scatter plot showing the association between log10-transformed pretreatment NLR and baseline BVAS. The solid line represents the fitted linear regression. BVAS, Birmingham Vasculitis Activity Score; NLR, neutrophil-to-lymphocyte ratio.

## Supplementary Figure 4. BVAS and GC dose over time according to pretreatment NLR

Boxplots show observed values at each time point. BVAS, Birmingham Vasculitis Activity Score; GC, glucocorticoid; NLR, neutrophil-to-lymphocyte ratio; ns, not significant.

**Supplementary Figure 5. Intergroup differences in the Vasculitis Damage Index**

NLR, neutrophil-to-lymphocyte ratio; ns, not significant; VDI, Vasculitis Damage Index.

**Supplementary Figure 6. Sensitivity analysis of intergroup differences in BVAS and GC dose among patients completing at least 12 months of follow-up**

Intergroup differences in BVAS and GC dose were analysed in patients who completed at least 12 months of follow-up. BVAS, Birmingham Vasculitis Activity Score; GC, glucocorticoid; NLR, neutrophil-to-lymphocyte ratio; ns, not significant.

**Supplementary Figure 7. Sensitivity analyses of landmark survival**

Landmark analyses using alternative landmark times of 2 and 3 years yielded findings consistent with those obtained using the 2.5-year landmark. NLR, neutrophil-to-lymphocyte ratio.

**Supplementary Figure 8. Association between pretreatment NLR, modelled as a continuous variable, and infection-related mortality**

Log₁₀-transformed pretreatment NLR was modelled using a restricted cubic spline with three knots in a univariable cause-specific Cox proportional hazards model. The solid blue line represents the estimated hazard ratio, and the blue shaded area represents the pointwise 95% confidence interval. Grey bars show the distribution of pretreatment NLR, with frequencies indicated on the right-hand axis. *P* values for the overall association and non-linearity are shown.

NLR, neutrophil-to-lymphocyte ratio.

**Supplementary Figure 9. Residual diagnostic plots for the univariable cause-specific Cox proportional hazards model using a restricted cubic spline**

(A) Martingale residuals plotted against pretreatment NLR on its original scale. (B) Scaled Schoenfeld residuals plotted against follow-up time to assess the proportional hazards assumption.

NLR, neutrophil-to-lymphocyte ratio.

**Supplementary Figure 10. Causes of infection-related death**

Distribution of infection-related causes of death according to pretreatment NLR tertiles. CMV, cytomegalovirus; COVID-19, coronavirus disease 2019; NLR, neutrophil-to-lymphocyte ratio.

**Supplementary Figure 11. Covariate balance before and after inverse probability weighting**

Love plot showing absolute standardised mean differences before and after stabilised inverse probability weighting.

BVAS, Birmingham Vasculitis Activity Score; GC, glucocorticoid; mPSL, methylprednisolone; IVCY, intravenous cyclophosphamide.

**Supplementary Figure 12. Sensitivity analysis of weighted infection-related mortality-free survival**

Shaded areas indicate 95% confidence intervals. Pairwise *P* values were adjusted for multiple comparisons using the Bonferroni method. The tables below the curves show weighted numbers at risk and weighted cumulative event counts.

**Supplementary Figure 13. Covariate balance before and after inverse probability weighting for sensitivity analysis**

Love plot showing absolute standardised mean differences before and after stabilised inverse probability weighting of sensitivity analysis.

BVAS, Birmingham Vasculitis Activity Score; eGFR, estimated glomerular filtration rate.

**Supplementary Figure 14. Diagnostic plots based on scaled Schoenfeld residuals for the inverse-probability-weighted Cox models**

The proportional hazards assumption was statistically assessed using scaled Schoenfeld residuals.
